# Supplementary material for: Chromothripsis during telomere crisis is independent of NHEJ, and consistent with a replicative origin
Source: Genome Res. 2019 May;29(5):737–49. doi: 10.1101/gr.240705.118 (PMC6499312; doi:10.1101/gr.240705.118)
Supplement: Supplemental Material [file supp_gr.240705.118_Supplemental_file_1.zip › contigs/annotated_contigs/DB51/contig.2.DB51_length_812_mean_cov_6.28325123153.docx]

**DB51_length_812_mean_cov_6.28325123153**

```
AATGTTGAAAACAGAGTTCACTCCCAGGTTGGGTCTACTTGAGCCAGACAGTCATTGTCAAAAATCACATGCCCCAAATGGGCAGAGTT
 >chr18:60833087-60833428 + E=3e-190
CGTTTGCTGGATCGCTTTAAAGCTGTGCCGTCTGCCTTGAAGCAAGTCCACTGTCATTTTGTTAAACAAGGGTCCTTCATCCAATACTG

GTTATGGCCAGGGTTGCAGACCCACAGGACACAAAGCCTGGCCCTCAGTATGAGGACCAGCTGCTGGCTGGCTTCCTCGGAGGGGGACT

GTGGGCTTGGGGGGCACTCACGGCTTTATAGACTGCCCAGTCCCATGCCCCAGAGAGAGGTGGTTGGCAGGA|AG|GATCTTAGTGTTT
                                                                         >chr10:2954673
TCAGGAGAAAAATGAATGAATGAACTAATCCCCTTCTGGAGAAAACTCAGGATGTGTTGATGACTGGAACTCCTTGTCAACTTTTCCCA
4-29547202 + E=1e-269
GATGCTTTCATGAGTTCGAGGTCAATCTTTTCTGGGAACAGTCCAGCAATTTCACCCCAAGTGTCAACTTTGTAAAAATACTCGGTAGA

CAAGAATCCAGGAATGGTTCCTTAATCTATGGCACCCAAGGCCAAGAGAGCAGACAGTTTTCCCAGGCTGCTCAGGGTTGACTTTACTA

AACCGACCATAATTCATACTAACACCTTGAATAATGAGGAGCTCTAAACACATGAGAAAATCCAATAAAGTATTTTAGAAGACAAACAA

GAAGACTAGCAGTAGACTTGCAGTGCCAGGTGCTATTACAAAGCTGCAGTAATTAAACGAATATGCCAGTAGCCCCAGAATGGACAAAG

AAGTCAAT|AAAAA
```
